# Supplementary material for: Adequacy of using a single nasal swab for rapid influenza diagnostic testing, PCR, and whole genome sequencing
Source: PLOS Glob Public Health. 2023 May 24;3(5):e0001422. doi: 10.1371/journal.pgph.0001422 (PMC10208452; doi:10.1371/journal.pgph.0001422)
Supplement: Attachment — Submitted filename: Reviewer Response (002)_RG edits-jlt_2-23-22.docx [file pgph.0001422.s001.docx]

Dear Dr. Croda,

**Thank you for considering our manuscript, “Adequacy of using a residual nasal swab specimen from a rapid influenza diagnostic test to perform PCR and whole genome sequencing.” We are grateful for the opportunity to make revisions based on reviewer feedback. The following is our attempt to address the concerns detailed in your email.**

**EDITOR COMMENTS**

1. Please send a completed 'Competing Interests' statement, including any COIs declared by your co-authors. If you have no competing interests to declare, please state "The authors have declared that no competing interests exist". Otherwise please declare all competing interests beginning with the statement "I have read the journal's policy and the authors of this manuscript have the following competing interests:"

**The declaration of competing interest statement has been revised to include the requested language.**

2. Please amend your detailed Financial disclosure statement. This is published with the article. It must therefore be completed in full sentences and contain the exact wording you wish to be published.
   a. State the initials, alongside each funding source, of each author to receive each grant.
   b. State what role the funders took in the study. If the funders had no role in your study, please state: “The funders had no role in study design, data collection and analysis, decision to publish, or preparation of the manuscript.”
   c. If any authors received a salary from any of your funders, please state which authors and which funders.

If you did not receive any funding for this study, please simply state: “The authors received no specific funding for this work.”

**The financial disclosure statement has been revised to include the requested information.**

3. We do not publish any copyright or trademark symbols that usually accompany proprietary names, eg (R), (C), or TM  (e.g. next to drug or reagent names). Please remove all instances of trademark/copyright symbols throughout the text, including R and TM on page 8 and 9.

**Copyright and trademark symbols have been removed.**

4. In the online submission form, you indicated that your data will be submitted to a repository upon acceptance.  We strongly recommend all authors deposit their data before acceptance, as the process can be lengthy and hold up publication timelines. Please note that, though access restrictions are acceptable now, your entire data will need to be made freely accessible if your manuscript is accepted for publication. This policy applies to all data except where public deposition would breach compliance with the protocol approved by your research ethics board. If you are unable to adhere to our open data policy, please kindly revise your statement to explain your reasoning and we will seek the editor's input on an exemption. Please be assured that, once you have provided your new statement, the assessment of your exemption will not hold up the peer review process.

**Data has been published and can be found at the following location:** [**https://doi.org/10.7910/DVN/RLXPUM**](https://doi.org/10.7910/DVN/RLXPUM)

**REVIEWER #1**

The authors measured the accuracy of rapid influenza diagnostic testing of anterior nasal swabs compared to RT-PCR from nasopharyngeal or oropharyngeal (NP/OP) swab specimens. They selected 199 participants from a previous influenza prospective surveillance study and measured the accuracy of rapid diagnostic tests using NP/OP RT-PCR results as the gold standard of positivity. This is an important applied public health question. I have several questions about the methods, in particular about WGS methods and results, that should be addressed.

**We apologize for any confusion, but the purpose of this manuscript was not to measure the accuracy of rapid influenza diagnostic testing of anterior nasal swabs compared to NP/OP swabs, nor the accuracy of rapid diagnostic tests. Those points are fairly well established in the literature, and we have already contributed to the discussion.^1-3^ The purpose of this study was to see if it’s reasonable to use a single nasal swab for more than one test (i.e. rapid antigen testing, PCR confirmation of rapid results, and whole genome sequencing). This is a topic that has become increasingly important throughout the pandemic due to widespread use of rapid antigen tests and supply shortages.**

1. Abstract. Please define what is meant by “successful” WGS. Is this based on a pre-specified coverage depth and breadth threshold?

**To address the confusion around the term “successful” we have revised the sentence to say, “Genomic information was extracted from all 40 rNS specimens and 37 of the 40 NP/OP specimens. Complete WGS reads were available for 67.5% (14 influenza A; 13 influenza B) of the rNS specimens and 59.5% (14 influenza A; 8 influenza B) of the NP/OP specimens.”**

2. Methods. Please also include a section on whole genome sequencing, including library preparation, sequencing, and bioinformatic analysis.

**The following language has been added to the methods section:** **“Next-generation whole-genome sequencing was performed using multisegment reverse transcription-PCR (M-RTPCR) with Invitrogen Super-Script III One-Step RT-PCR with Platinum Taq High Fidelity enzyme (Thermo Fisher Scientific, Inc ) to simultaneously amplify eight genomic RNA segments of influenza A and B viruses.^4,5^ Indexed sequencing libraries were generated from the M-RTPCR amplicons using the Nextera XT Sample Preparation Kit ( Illumina, San Diego, CA, USA) and sequenced on the MiSeq platform using MiSeq Reagent Kit v2 (300-cycles) reagents. Raw sequencing reads were analyzed using the IRMA approach and consensus sequences were manually checked to verify data quality.^6^”**

3. Methods. How did recruitment work? Was there informed consent and IRB approval? Was this study exempt?

**Recruitment was up to clinician discretion as long as a patient met the inclusion criteria. The following line has been added to the first paragraph of the methods section: “The University of Wisconsin Health Sciences and Minimal Risk Research Institutional Review Board classified W-IISP as part of routine care and considered exempt.”**

4. Results. Please expand the paragraph on whole genome sequencing. As written, is difficult to understand what is meant by complete WGS reads and what is meant to “WGS was successfully performed.”

**Per your request, we have expanded description of WGS to include additional details on incomplete vs complete genomic reads and revised the language to provide additional clarity. The last paragraph in the results section was replace with the following two paragraphs:**

**“In the subset of the 40 paired specimens, three NP/OP specimens were excluded due to insufficient volume. For the remaining 77 specimens, 27 of the 40 rNS specimens (67.5%; 14 influenza A and 13 influenza B) and 22 of the 37 NP/OP specimens (59.5%; 14 influenza A and 8 influenza B) produced complete genomic information. To be considered complete, all segments needed to be assembled (8/8 influenza genes) and pass a quality control test. For the 28 incomplete specimens, 16 were unable to assemble 1 segment, 7 were unable to assemble 2 segments, and 5 were unable to assemble 3 or more segments.**

**Specimens with a complete genome had a mean Ct of 23.38 (+/- 3.00) and specimens with an incomplete genome had a mean Ct of 26.36 (+/- 4.26; t=-3.58; p <0.001). The mean Ct values for the 20 influenza A and 20 influenza B rNS specimens were 24.75 [range: 21.08—28.67] and 24.76 [range: 19.04—28.59], respectively. The mean Ct values were similar between the 40 rNS specimens and 37 NP/OP specimens (24.8 vs. 24.7). The differences in mean Ct values were not statistically significant regardless of virus or swab type.”**

5. Results. Was Ct associated with coverage? Did coverage differ between viral types?

**Ct value was associated with coverage and did not differ between virus types. See above for updated language in the results section.**

6. Results. Were the WGS data deposited to a publicly available sequence repository?

**These data were not uploaded to a public database.**

7. Discussion. I’d suggest including that the rNS and NP/OP Cts were not significantly different (if that is the case), rather than the percentage of rNS with lower Ct values.

**We added the following language to the discussion section: “There was no significant difference in the mean Ct values for rNS and NP/OP specimens.”**

**REVIEWER #2**

1. Tables need legends.

**We are unclear as to what legends are needed, but we spelled out PPV and NPV in Table 3 in case that was the concern.**

2. Did not have any exclusion criteria?

**Recruitment was up to clinician discretion as long as a patient met the inclusion criteria. Specimens were excluded from the analysis if the Ct value was 29 or greater. We added a flow chart to clarify the data selection process.**

3. “RT-PCR was performed on NP/OP swabs within 1 to 9 days of specimen collection (mean = 3.03 days; median = 3 days).” Were the NP and OP samples sent together with the rNS? It was not well described what was done with the NP and OP swabs. It just says RT-PCR was done within 1 to 9 days.

**In the paragraph before the quoted sentence, we explained that the rNS and the NP/OP specimens were packed together and sent via courier to the state laboratory. At the state lab, an aliquot of the rNS was frozen and then tested after the influenza season concluded. The NP/OP specimen was never frozen and tested within 9 days of specimen collection. We made minor edits that we hope will clarify the process.**

4. “Between July 1, 2014 and June 30, 2015, 962 paired specimens were collected from 986 patient encounters (97.6% dual swab compliance). Nasal swabs were not collected for 21 cases; NP/OP swabs were not collected for 3 cases. RT-PCR detected 248 (25.8%) cases of influenza—166 (17.3%) influenza A (H3N2), 4 (0.4%) unsubtypeable influenza A, and 78 (8.1%) influenza B. The unsubtypeable specimens were removed from further analyses. The remaining 958 specimens were separated into three groups from which specimens were randomly selected using the random sample application in Minitab to produce a set of 200 specimens for validation testing (Table 1).” It was not evident during the reading that four of the 962 samples were removed, leaving 958. Perhaps remove these 4 from the table, put an asterisk in local specimens, and put in the legend that they were removed because it was impossible to subtype

**We added a flow chart to clarify the data selection process.**

**REFERENCES**

1.	Arnold MT, Temte JL, Barlow SK, et al. Comparison of participant-collected nasal and staff-collected oropharyngeal specimens for human ribonuclease P detection with RT-PCR during a community-based study. *PLoS One*. 2020;15(10):e0239000. doi:10.1371/journal.pone.0239000

2.	Bell C, Birstler J, Goss MD, et al. Factors influencing sensitivity of a rapid influenza diagnostic test in a community-based population of kindergarten through 12th-grade students: Wisconsin 2015-2020. *Influenza and other respiratory viruses*. Jan 2023;17(1):e13064. doi:10.1111/irv.13064

3.	Bell C, Goss M, Birstler J, et al. Assessment of potential factors associated with the sensitivity and specificity of Sofia Influenza A+B Fluorescent Immunoassay in an ambulatory care setting. *PLoS One*. 2022;17(5):e0268279. doi:10.1371/journal.pone.0268279

4.	Zhou B, Lin X, Wang W, et al. Universal influenza B virus genomic amplification facilitates sequencing, diagnostics, and reverse genetics. *J Clin Microbiol*. May 2014;52(5):1330-7. doi:10.1128/JCM.03265-13

5.	Zou XH, Chen WB, Zhao X, et al. Evaluation of A Single-reaction Method for Whole Genome Sequencing of Influenza A Virus using Next Generation Sequencing. *Biomed Environ Sci*. Jan 2016;29(1):41-6. doi:10.3967/bes2016.004

6.	Shepard SS, Meno S, Bahl J, Wilson MM, Barnes J, Neuhaus E. Viral deep sequencing needs an adaptive approach: IRMA, the iterative refinement meta-assembler. *BMC Genomics*. Sep 5 2016;17(1):708. doi:10.1186/s12864-016-3030-6
